# *Plasmodium falciparum *PfA-M1 aminopeptidase is trafficked via the parasitophorous vacuole and marginally delivered to the food vacuole

**DOI:** 10.1186/1475-2875-9-189

**Published:** 2010-06-30

**Authors:** Omid Azimzadeh, Cissé Sow, Marc Gèze, Julius Nyalwidhe, Isabelle Florent

**Affiliations:** 1 HelmholtZentrumMünchen, German Research Centre for Environmental Health, D-85764, Neuherberg, Germany; 2 FRE3206 CNRS/MNHN, Department Regulations, Development, Molecular Diversity, CP52, 61 rue Buffon, F-75005 Paris, France; 3 Department of Microbiology and Molecular Cell Biology, Eastern Virginia Medical School, Norfolk, Virginia, 23507 USA

## Abstract

**Background:**

The *Plasmodium falciparum *PfA-M1 aminopeptidase, encoded by a single copy gene, displays a neutral optimal activity at pH 7.4. It is thought to be involved in haemoglobin degradation and/or invasion of the host cells. Although a series of inhibitors developed against PfA-M1 suggest that this enzyme is a promising target for therapeutic intervention, the biological function(s) of the three different forms of the enzyme (p120, p96 and p68) are not fully understood. Two recent studies using PfA-M1 transfections have also provided conflicting results on PfA-M1 localization within or outside the food vacuole. Alternative destinations, such as the nucleus, have also been proposed.

**Methods:**

By using a combination of techniques, such as cellular and biochemical fractionations, biochemical analysis, mass-spectrometry, immunofluorescence assays and live imaging of GFP fusions to various PfA-M1 domains, evidence is provided for differential localization and behaviour of the three different forms of PfA-M1 in the infected red blood cell which had not been established before.

**Results:**

The high molecular weight p120 form of PfA-M1, the only version of the protein with a hydrophobic transmembrane domain, is detected both inside the parasite and in the parasitophorous vacuole while the processed p68 form is strictly soluble and localized within the parasite. The transient intermediate and soluble p96 form is localized at the border of parasitophorous vacuole and within the parasite in a compartment sensitive to high concentrations of saponin. Upon treatment with brefeldin A, the PfA-M1 maturation is blocked and the enzyme remains in a compartment close to the nucleus.

**Conclusions:**

The PfA-M1 trafficking/maturation scenario that emerges from this data indicates that PfA-M1, synthesized as the precursor p120 form, is targeted to the parasitophorous vacuole *via *the parasite endoplasmic reticulum/Golgi, where it is converted into the transient p96 form. This p96 form is eventually redirected into the parasite to be converted into the processed p68 form that is only marginally delivered to the parasite food vacuole. These results provide insights on PfA-M1 topology regarding key compartments of the infected red blood cells that have important implications for the development of inhibitors targeting this plasmodial enzyme.

## Background

Human malaria is the most important parasitic disease in the tropical countries in terms of morbidity and mortality [1]. The disease is caused by obligate intracellular protozoan parasites belonging to the genus *Plasmodium *and the species *Plasmodium falciparum *is responsible for the most severe forms of the disease and most of the mortality.

To maintain their intracellular mode of life in erythrocytes, *Plasmodium *spp. express a number of proteases that are involved in invasion processes, the acquisition and digestion of nutrients from the host cell, and that facilitate the exit of the parasite at the end of its intra-erythrocytic stage of development [2,3]. These proteins with different molecular functions, substrate specificities, and different cellular localizations have different physiological requirements for their activities. The *P. falciparum *zinc aminopeptidase, PfA-M1, displays a neutral aminopeptidase activity with an optimal activity at pH 7.4, and remaining at least 40% active from pH 5.8 to pH 8.6 [4]. Initially described in the trophozoite and schizont stages of the FcB1 strain of *P. falciparum*, PfA-M1 was shown by immunofluorescence studies to have a changing localization pattern during the course of the parasite development. In trophozoites, the PfA-M1 labelling was diffuse in the parasite cytoplasm with accumulations outside the digestive vacuole. In schizonts, it turned progressively to a vesicle-like pattern ending as a clear spot in released merozoites [4]. PfA-M1 was, therefore, thought to be involved either in the terminating steps of haemoglobin breakdown, that have been shown to take place outside the food vacuole [5], or in the egress from red blood cell/reinvasion process [4]. Encoded by a single copy gene on chromosome 13, PfA-M1 was first described to be a 1,056 amino acid enzyme displaying canonical signatures of the M1 family of metallopeptidases and a putative microbodies targeting signal at its C-terminus [6]. However, the released *P. falciparum *genome [7] predicted a gene model proposing that this protein also has a sequence extension at its N-terminus, which includes a putative N-terminal hydrophobic domain (see MAL13P1.56 in the PlasmoDB database and EMBL Y09081.2). Whether this hydrophobic domain behaves like a signal sequence or a signal anchor has never been addressed experimentally and may not be clearly predicted since different algorithms have yielded conflicting predictions [8-11]. Protease inhibitor treatment or exclusion during parasite harvest, protein isolation and purification, and the use of polyclonal antibodies specific for two peptide domains (MAP1 and MAP2) deduced from the gene, have detected three soluble forms of this enzyme designated p120, p96 and p68, respectively [4,6] (Additional file 1). Although all three forms contain the complete active site, the aminopeptidase activity could only be experimentally associated with pure p96 and pure p68 forms, because the p120 form could only be isolated in presence of protease inhibitors [4]. Conversely, the p96 form was obtained exclusively from parasites prepared in absence of protease inhibitors, and was, therefore, proposed to be an *in vitro *degradation product of p120 [4]. In parasites released after saponin lysis of infected red blood cells (iRBCs) in the presence of protease inhibitors, both the p120 and the p68 forms were identified and equivalent amounts of p120/p68 were found in both trophozoites and schizonts; early rings (six hours post-invasion) being devoid of either form of PfA-M1 [4]. The p120 and p68 forms were then proposed to be the two native forms of PfA-M1, both found in saponin-isolated parasites. These results did not conclusively address the question on how PfA-M1 biosynthesis and maturation occurs during the intra-erythrocytic cycle of *P. falciparum*, since two cleavages - one upstream and one downstream of the enzyme active site - are necessary to yield p68 from p120 (see Additional file 1). These results also left open the question as to whether p120, p96, and p68 played any distinct functional role in the biology of the parasite [4].

In this report, the biosynthesis and maturation of PfA-M1 during the *P. falciparum *erythrocytic cycle was further investigated by focusing on the presence of the different forms in distinct compartments of the infected red blood cells.

## Methods

### Materials

Streptolysin O (SLO) was a kind gift from Sucharit Bhakdi (University of Mainz, Germany). Saponin was purchased from Fluka. Sequence-grade modified trypsin was purchased from Promega (Madison, WI, USA). All the other chemicals, including Brefeldin A (BFA), were of the highest available purity and were purchased from Sigma-Aldrich.

### Parasite cultures and fractionation of iRBC

Parasites (FCBR strain of *P. falciparum *[12]) were continuously cultured in erythrocytes of blood group A^+ ^in heat inactivated human serum as described previously [13]. Cultures were synchronized and trophozoite infected erythrocytes (iRBC), 28-30 hours post infection, were enriched to a parasitaemia of more than 90% by gel flotation [14]. IRBC were permeabilized with SLO as described previously [15]. Briefly, 1 × 10^9 ^iRBC (in aliquots of 2 × 10^8 ^cells) were incubated with 3-4 haemolytic units of SLO in PBS pH 7.2 at room temperature for 6 min. Samples were centrifuged at 10,000 × g for 15 s. The pellet containing intact parasites, the vacuolar contents, and membranes was washed twice with 200 μl of PBS. The removal of haemoglobin was monitored spectrophotometrically as described previously [15]. For saponin lysis, 1 × 10^9 ^iRBC (in aliquots of 2 × 10^8 ^cells) were incubated in 200 μl of 0.1% saponin in PBS pH 7.2 on ice for 5 min. The samples were centrifuged at 2,500 × g for 5 min. The pellet containing the intact parasite devoid of the host cytosol and PV contents was washed twice with 200 μl of PBS before processing for further analysis by SDS-PAGE and western blot. To prevent proteolysis by endogenous proteases, all the buffers used in this section contained a protease inhibitor cocktail (PIC) consisting of antipain, chymostatin, aprotinin, trypsin inhibitor, Na-EDTA, pepstatin, leupeptin, and elastatinal, each at a concentration of 1 μg.ml^-1^.

### Stage specific expression experiments

Parasite cultures (FCBR strain) were synchronized and trophozoite-infected erythrocytes (iRBC), 28-30 hours post-infection, were enriched to a parasitaemia of more than 90% by gel flotation [14]. Aliquots equivalent to 2 × 10^8 ^iRBC were used to initiate new 5 ml cultures. After reinvasion these cultures were synchronized by alanine treatment [16] to obtain tightly synchronized parasite stages. The parasites were harvested 6-12 hours, 18-24 hours, 30-36 hours and 38-44 hours post-invasion. 2 × 10^7 ^equivalent amounts of the parasites from each set time point were lysed in SDS-PAGE sample buffer before analysis by SDS-PAGE and western blot. All the buffers used in this section contained the protease inhibitor cocktail (PIC).

### Recombinant p68 form and production of specific anti-p68 antibodies in mice

The region encoding the p68 form of PfA-M1 was amplified by using Forward 5'-AC**G GAT CC**T GTT AAA AAG AAC GAA CC-3' and Reverse 5'-AT**G GAT CC**A TTG TGC ATT TAC TGG TG-3' primers (*BamH*I site in bold and underlined) and inserted into the *BamH*I site of pET-15b (Novagen). The recombinant plasmid was sequenced to confirm the correct reading frame and the absence of mutation. It encodes a N-terminal (His)_6_-tagged protein, corresponding to residues 191 to 802 of PfA-M1. This (His)_6_-p68 recombinant protein was produced in BL21(DE3) *Escherichia coli *as described [17], but was not soluble. It was isolated, by using BugBuster™ (Novagen) in presence of 6 M urea according to manufacturer's instructions and purified on nickel-affinity columns, in presence of urea. The eluted fractions containing recombinant (His)_6_-p68 were dialyzed against 10 mM HEPES NaOH pH 8.0 to remove urea and concentrated on Centriprep10 (Ultrafiltration device, Millipore) prior to being used to immunize three mice. Mice were injected four times with the purified-recombinant (His)_6_-p68 at three weeks intervals, the first injection being performed with complete Freund's adjuvant and the next three injections with incomplete Freund's adjuvant. The three mice produced antibodies specifically labelled p68, but also p96 and p120, as determined by immunoblotting.

### 2D-gel electrophoresis for the comparative analysis of anti-MAP1 and anti-p68 antibodies

Parasites (FcB1 strain of *P. falciparum*) were cultured but not synchronized, and isolated by using 0.1% saponin as previously described [4]. Purified parasites were lysed in 0.1 mM Tris-HCl pH 7.5 by three cycles of freezing/thawing in presence of protease inhibitors (Complete™ EDTA-free (Boehringer Mannheim) and 2 mM EDTA) as previously described [4], and separated into soluble and membrane fractions by ultra-centrifugation (100,000 × g for one hour). 100,000 g soluble extracts were concentrated by using kit-2D clean up (GE Healthcare), and identical amounts (70 μg proteins) were used to rehydrate two identical 3-10 strips (GE Healthcare) in the presence of 8.75 M urea, 2.5 M thiourea, 5% CHAPS, 3.5 mg.ml^-1 ^DTT and 2% ampholytes (GE Healthcare). After electrofocusing, the two strips were equilibrated 15 minutes in 6 M urea, 50 mM Tris-HCl pH 8.8, 70 mM SDS, 34.5% Glycerol and 65 mM DTT then 15 minutes in 6 M urea, 50 mM Tris-HCl pH 8.8, 70 mM SDS, 34.5% Glycerol and 130 mM iodoacetamide, before being placed side by side on a single large 7%-SDS-PAGE. The SDS-PAGE was run and processed for immunoblot analysis, as described in the corresponding section.

### Brefeldin A treatment of parasites

Parasites (FcB1 strain of *P. falciparum*) were cultured and synchronized as previously described [4] in the presence of 367 μM hypoxanthine to obtain a population of early rings (one to six hours post-invasion) that was divided into three identical cultures (B, E and O, with about 5 × 10^8 ^iRBC each). Brefeldin A (BFA) was added to B at a final concentration of 5 μM (about 1.5 μg.ml^-1^) from a 5 mg.ml^-1 ^solution in ethanol as previously described [18]. Control cultures received (E) or not (O) equivalent amount of ethanol to ensure that ethanol had no effect on growth and cell morphology. These three cultures were further incubated for 19 hours prior to parasite harvest and preparation by using 0.1% saponin as described [4]. Purified parasites were lysed in 0.1 mM Tris-HCl pH 7.5 by three cycles of freezing/thawing and analysed by SDS-PAGE and immunoblotting as described in the corresponding section.

### Immunofluorescence analysis of parasites

In parallel, aliquots of BFA-treated and control cultures were smeared on slides and fixed for 5 min in a mixture of methanol and acetone (1:4) at -20°C. Slides were washed in PBS, blocked at room temperature in non-fat milk (5% in PBS) then incubated for two hours at room temperature with anti-MAP1 antibodies as previously described [4]. Slides were then washed again three times in PBS and incubated for two hours at room temperature with mouse anti-exp2 [19] antibodies. After three further washes in PBS, slides were incubated with a mixture of anti-rabbit-Ig coupled to Alexa-fluor-568 and anti-mouse-Ig coupled to Alexa-fluor-488 secondary antibodies (Molecular Probes). Nuclei were labelled by 10 min incubation with 4 μg.ml^-1 ^Hoechst 33342 (Molecular Probes). Slides were mounted in Vectashield medium (Vector laboratories) with a coverslip and viewed using a Nikon Eclipse TE 300 DV inverted microscope with a 100× (NA 1.3) oil objective mounted on a piezo electric device using appropriate fluorescence emission filters. Image acquisition was performed using a Coolsnap HQ camera (Roper Scientific, France) and images were finally processed by using Metamorph software.

### Isolation of intact food vacuoles

To isolate intact food vacuoles parasites were fractionated as previously described with minor modifications [3,20]. Briefly 10^9 ^iRBC were washed three times with PBS and permeabilized with SLO as described previously. The iRBC (in aliquots of 2 × 10^8 ^cells) were incubated with 3-4 haemolytic units of SLO in PBS pH 7.2 at room temperature for 6 min. Samples were centrifuged at 10,000 × g for 15 s. The pellet containing intact parasites, the vacuolar contents, and membranes was washed twice with 200 μl of PBS. To release the food vacuoles the pellet was lysed by resuspension in 10 volumes of ice-cold water, pH 4.5, and immediately triturated four times using a 27-G 1.2 cm needle. The mixture was centrifuged at 13,000 rpm for 10 min to obtain the soluble proteins (A) and a pellet containing membranes and vacuoles. The crude vacuole preparation was washed with 10 volumes of ice-cold water, pH 4.5 centrifuged at 13,000 rpm for 2 min and the supernatant was analysed (B) to monitor the distribution of marker proteins. The pellet was resuspended in 1 ml of uptake buffer (2 mM MgSO_4_, 100 mM KCl, 10 mM NaCl, 25 mM HEPES, 25 mM NaHCO_3_, and 5 mM sodium phosphate pH 7.4, containing 5 mg.ml^-1 ^of DNase 1), and incubated at 37° C for 5 min followed by centrifugation for 2 min at 13,000 rpm. The supernatant was discarded and the pellet was again resuspended in 100 μL of ice-cold uptake buffer and mixed with 1.3 ml of ice-cold 42% Percoll™ containing 0.25 M sucrose and 1.5 mM MgSO4, pH 7.4. The suspension was triturated 2 times through a 27-G 1.2-cm needle before centrifugation at 13,000 rpm for 10 min at 4°C. The fractions C, D, E, and F were obtained after the centrifugation step. Purified vacuoles (E) were collected as a dark band at the bottom 50 μL of the gradient. The vacuoles were then resuspended in 1 mL of uptake buffer, and centrifuged at 13,000 rpm for 2 min to wash off the residual Percoll™ before proceeding with immunoblot analysis using an equivalent of 4 × 10^7 ^iRBC per lane. All the buffers used in this section contained the protease inhibitor cocktail (PIC).

### Western blot analysis

Proteins separated by SDS-PAGE were transferred to nitrocellulose membranes using standard procedures. To detect the presence of PfA-M1 [4,6] and the other marker proteins (PfAldolase [15], PfBip [21], PfPV1 [22], PfPlasmepsin I [23], PfSERP [18]), the membranes were blocked with 3% BSA in PBS, pH 7.4, for 1 h at room temperature before overnight incubation at 4°C with the primary antibodies. After the overnight incubation the membranes were washed three times with 10 mM Tris-HCl, pH 7.4, 150 mM NaCl before incubating with the secondary antibody, anti rabbit IgG conjugated with horseradish peroxidase (FCBR strain) or anti rabbit IgG conjugated with alkaline phosphatase (FcB1 strain) for one hour. The proteins bands were visualized using the ECL (FCBR strain) or nitroblue tetrazolium/5-bromo-4-chloro-3-indol phosphate (FcB1 strain).

### Stable transfection of *P. falciparum *with a PfA-M1-GFP construct

The plasmid pPM2GT, used to stably transfect plasmepsin II-GFP into *Plasmodium *[24], kindly provided by Dr. Klemba, was modified as follows: the plasmepsin II gene segment encoding the C-terminal end of the protease was excised by using the *Avr*II and *Xho*I sites and was replaced by a 1024-bp segment encoding the C-terminal part of PfA-M1. To amplify this PfA-M1 gene segment, we used the Forward 5'-GCA CG**C TCG AG**T AAT TAT TAT TGA AAT ATG ATA GTG ATG C-3'(*Avr*II site in bold) and Reverse 5'-GCA CG**C CTA GG**T AAT TTA TTT GTT AAT CTT AAT AAA TAT TC-3'(*Xho*I site in bold) primers. The recombinant plasmid was sequenced to confirm the correct reading frame and the absence of mutation before transfection into the FcB1 strain of *P. falciparum *according to [25]. Transfected parasites were selected using 10 nM WR99210 as described in [24] and the selective pressure was removed and applied again to isolate pseudo-clonal parasites named PfA-M1-GFP-51. These parasites, which are very similar to the PfA-M1-YFP parasites generated by [26] were analysed by southern blotting to confirm proper integration of the plasmid, immunoblotting to visualize the recombinant protein and fluorescence imaging to localize the GFP fluorescence. Live imaging was performed on parasites stained with 4 μg.ml^-1^Hoechst 33342, using the Nikon Eclipse TE 300 DV inverted microscope, the Coolsnap HQ camera and the Metamorph software for image processing, as described above for the immunofluorescence imaging.

### Transient transfection of *P. falciparum *with a PfA-M1 [1-30]-GFP construct

The PfA-M1 [1-30]-GFP plasmid was constructed using Multisite Gateway™ technology described by van Dooren *et al *[27]. Briefly, the Multisite Gateway™ is based on specific recombination of pENTR plasmids called promoter vector, gene vector and reporter vector with a destination vector. Promoter vector (PfHSP86), reporter vector (GFP mut2) and destination vector (hDHFR cassette conferring resistance to WR99210 [28]) were kind gifts of G. van Dooren and G. MacFadden. The gene vector specific for the first 30 amino acids of PfA-M1 was constructed by amplifying the corresponding segment (on *P. falciparum *genomic DNA) using Forward 5'-CAC CAT TAC AAA ATG AAA TTA ACA AAA GGC TG-3' and Reverse 5'-GCA CCT TTT TTT ATT ATC ATA AAG AAT-3'primers and inserting the segment into pENTR™D/Topo^® ^(Invitrogen). The recombinant plasmid was produced following the Multisite Gateway™ technology (Invitrogen) as described [27] and sequenced to confirm the correct reading frame and the absence of mutation. *P. falciparum *(FcB1 strain) parasites were then transfected with this plasmid and transfectants were selected using 5 nM WR99210 as described [27]. Transfectants were analysed by immunoblotting and live imaging as described above.

## Results

### Antibodies raised to the p68 form of PfA-M1 confirm the three p120, p96 and p68 forms of PfA-M1

PfA-M1 was previously reported to be present in three soluble forms named p120, p96, and p68 by using two anti-peptide antibodies anti-MAP1 and anti-MAP2 [4]. To confirm that no additional form of PfA-M1 exists in the parasite, antibodies were raised against a recombinant protein corresponding to the p68 form and were used to immunoblot parasite extracts separated by 1D or 2D-gel electrophoresis (Additional file 2). Identical patterns are revealed using either anti-p68 or anti-MAP1 antibodies, thus confirming that these three forms are the only PfA-M1 soluble forms found in *P. falciparum *red blood cells. Moreover, immunoblot analyses of parasite membrane fractions indicated that among these three forms, only p120 was, in part, associated with membrane fractions while p96 and p68 were strictly soluble (see below).

### The p120 form of PfA-M1 is expressed in ring stages at least 12 hours before p68 is detected

**Figure 1:**
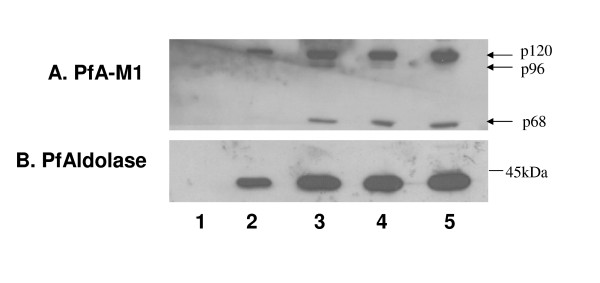
**PfA-M1 is expressed under the p120 form before the apparition of p96 and p68**. The expression of the p120, p96, and p68 forms of PfA-M1 is stage specific. Soluble parasite extracts from: non infected erythrocytes (lane 1); infected erythrocytes 6-12 hours post invasion (lane 2); infected erythrocytes 18-24 hours post invasion (lane 3); infected erythrocytes 30-36 hours post invasion (lane 4); infected erythrocytes 38-44 hours post invasion (lane 5), were blotted with anti-MAP1 antibodies [6] to detect PfA-M1 (A) and anti-Pf Aldolase antibodies (B) [15] as control. The p120 is expressed early in the erythrocytic stages and it is detectable at 6-12 hours post invasion (lane 2). The amount of p120 increases to a maximum at 38-44 hours post invasion (lane 5). In contrast p68 is not detected 6-12 hours post invasion (lane 2) but is appearing 18-24 hours post invasion (lane 3). Traces of p96 are observed at 18-24 hours and 30-36 hours post invasion (lanes 3 and 4).

PfA-M1 was previously shown to be expressed in trophozoites and schizonts with no expression in early rings (0-6 hours post-invasion) [4], but its transcriptomic profiles later suggested that it could be expressed in more mature rings [29,30]. A time course study was performed indicating not only that PfA-M1 was indeed expressed as early as 6-12 hours (post invasion) but that, at this stage, only the p120 form was found in parasites (Figure 1, lane 2). The p68 form was absent. At 18-24 hours post infection, both the p120 and the p68 forms were present and these persisted to the end of the cycle, in agreement with the earlier observations [4]. Traces of p96 were also detected at 18-24 and 30-36 hours post infection (Figure 1, lanes 3 and 4). These results indicate that p120 is expressed in parasites before the appearance of p96 and p68, and the less abundant form of p96 is mainly observed in mid-stages parasites.

### PfA-M1 is also present in the parasitophorous vacuole

A proteomic study of the parasitophorous vacuole (PV) was previously performed revealing numerous proteases and chaperones but not PfA-M1 [22]. In further analyses, the PV proteome has been further investigated by using other more sensitive proteomics approaches including Difference In Gel Electrophoresis (DIGE) after differential fractionation of infected erythrocytes with streptolysin O, saponin, and LC-MS-MS. Using this DIGE approach and peptide mass fingerprinting it was possible to identify PfA-M1 with high sequence coverage, and the identified peptides covered almost the entire length of the protein with the exception of the N-terminal hydrophobic domain and the C-terminus of the protein (J. Nyalwidhe, personal communication). These data suggested that PfA-M1 is present in the PV lumen.

**Figure 2:**
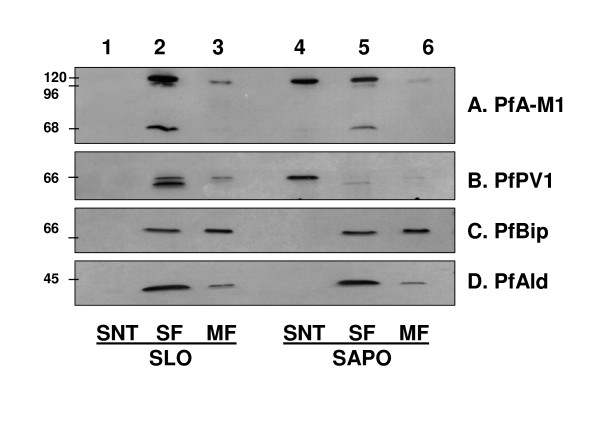
**The p120 form of PfA-M1 is present in the PV space**. Identical amounts of synchronized trophozoite stages were treated with streptolysin O (SLO) (lanes 1, 2, 3) or 0.1% saponin (SAPO) (lanes 4, 5, 3) as described in the Methods section. Lane 1 corresponds to SLO supernatant (SNT) i.e. the cytoplasm of iRBC while lane 4 corresponds to SAPO supernatant, i.e. the cytoplasm of iRBC plus PV content. Remaining pellets containing intact parasites devoid of host cytosol (SLO treatment) or devoid of host cytosol plus PV content (SAPO treatment) were washed twice in PBS, further lysed in ice-cold water in presence of protease inhibitors and separated by centrifugation into soluble fractions (SF, lanes 2 and 5, for the SLO and SAPO treatments, respectively) and membrane fractions (MF, lanes 3 and 6, for the SLO and SAPO treatments, respectively). Processed samples were separated by SDS-PAGE and blotted with antibodies corresponding to PfA-M1 (A. anti-MAP1 antibodies [6]) and several control markers: anti-PfPV1 antibodies (B), used as PV markers [22], anti-PfBip (C) [21] and anti-PfAldolase (D) antibodies [15] used as parasite reticulum endoplasmic and cytoplasm markers, respectively. The molecular masses kDa are shown on the left.

To validate these observations and to determine which forms of PfA-M1 were present in the PV lumen, the pore forming toxin streptolysin O (SLO) and the detergent saponin (SAPO) were used on identical samples of synchronized trophozoite stage *P. falciparum *infected red blood cells (iRBC). SLO releases soluble erythrocyte components but leaves the parasitophorous vacuole membrane (PVM) intact while saponin disrupts the erythrocyte membrane and the PVM thus releasing the contents of the parasitophorous vacuole [15]. Using antibodies directed against marker proteins for defined compartments within the infected erythrocytes (PfPV1, a marker of parasitophorous vacuole [22]; PfBip, a marker of the parasite endoplasmic reticulum (ER) [21] and PfAldolase, a marker of the parasite cytosol; [15]) it was confirmed that SLO permeabilization did not affect the integrity of the PVM (the soluble proteins of the parasitophorous vacuole, PfPV1 is absent from lane 1, Figure 2B). In this assay, the control parasite PfBip and PfAldolase as well as PfA-M1 were not released into the supernatant fraction (they are absent from lane 1, Figure 2A, C, D). Conversely, the 0.1% saponin treatment released soluble proteins of the parasitophorous vacuole (such as PfPV1, lane 4, Figure 2B) without affecting internal parasite proteins (PfBip and PfAldolase, lane 4, Figure 2C, D). Interestingly, this 0.1% saponin treatment also released a proportion of p120 form of PfA-M1 (lane 4, Figure 2A) confirming its presence in the PV. The p68 form of PfA-M1 remained exclusively within the parasite, together with an internal population of the p120 (lane 5, Figure 2A). Note also that only p120 is, in part, associated with membrane fractions (Figure 2A, lanes 3 and 6). These results show that PfA-M1 is not translocated beyond the parasitophorous vacuole membrane to the cytoplasm of the infected red blood cell. In addition, PfA-M1 does not contain PEXEL, VTS, or the HTS sequences or motifs that have been proposed to be responsible for the targeting of parasite proteins across the PVM into the erythrocyte cytosol [31,32]. However, PfA-M1 appears to have the capacity to be exported or secreted into the parasitophorous vacuole, most likely by using its N-terminal hydrophobic domain which has been shown to act as a recessed signal sequence in some other *P. falciparum *proteins [33].

### Brefeldin A blocks the p120 form of PfA-M1 near the nucleus

**Figure 3:**
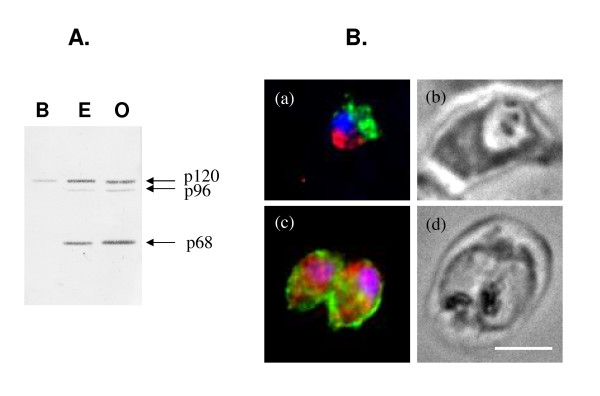
**BFA blocks the p120 form of PfA-M1 in a compartment close to the nucleus**. Synchronized parasites were treated for 19 hours in presence of 5 μM BFA (B) or in presence (E) or absence (O) of ethanol, in control cultures. **A**. Parasites were harvested, isolated by using 0.1% saponin and soluble fractions were blotted with anti-MAP1 antibodies to detect PfA-M1. The p120 form of PfA-M1 is present in BFA-treated cultures while p120, p96 and p68 forms are present in both control cultures, E and O. **B**. BFA-treated (a) and control parasites in presence of ethanol (c) were analysed by immunofluorescence as previously described [4,6] by using anti-MAP1 antibodies [6] to detect PfA-M1 (red) and a mouse anti-exp2 [19] serum as positive control (green). Nuclei, stained by using Hoechst 33342 appear in blue. Corresponding phase contrasts are in (b, d). Scale bar, 5 μm.

Brefeldin A (BFA) is a fungal metabolite that specifically inhibits anterograde transport from the ER to Golgi in many eukaryotic cells [34,35] and has been used as a tool to investigate protein trafficking in *P. falciparum *[19,33]. Here, BFA treatments were used to investigate PfA-M1 maturation and trafficking. Synchronized early ring stages were treated with 5 μM BFA for 19 hours before parasite harvest and analysis. The immunoblot analysis (Figure 3A) shows that in BFA-treated parasites only the p120 form is present in contrast to the control cultures grown in the presence (E) or absence (O) of ethanol, the solvent used to solubilize the BFA. In these latter cultures, as expected, p120, p96 and p68 forms were present, as previously observed [4]. The immuno-fluorescence analysis revealed that, in BFA treated parasites, PfA-M1 was retained in a compartment close to the nucleus that appears to be distinct from the compartment where exp-2 is retained upon BFA treatment (Figure 3B). Exp-2 is a protein exported to the PVM [19,36]. In the control culture, exp-2 was targeted as expected to the PVM and PfA-M1 was diffuse in the parasite (but outside the FV) as previously shown [4]. Therefore, it appears that BFA blocks the p120 form PfA-M1 in a compartment close to the nucleus that could correspond to the ER, and this blockage prevents its maturation to the p96 and p68 forms.

### The first 30 amino acids of PfA-M1 behave as a non-cleavable signal peptide

**Figure 4:**
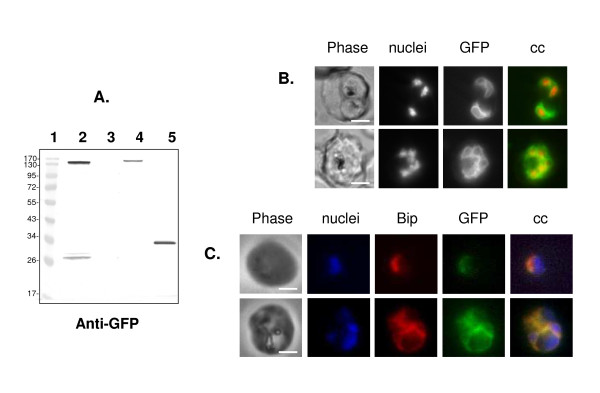
**PfA-M1 [1-30]-GFP yields a 32-kDa protein strictly associated with the parasite membranes that localizes to the ER**. **A**. Soluble proteins (lanes 2 and 3) and membrane proteins (lanes 4 and 5) from parasites expressing PfA-M1-GFP chimera (51, lanes 2 and 4) or PfA-M1 [1-30]-GFP chimera (lanes 3 and 5) were separated by SDS-PAGE, transferred to nitrocellulose and immunoblotted with anti-GFP antibodies as described in the Methods section. PfA-M1-GFP chimera was detected at ~150-kDa in soluble (lane 2) and membrane (lane 4) fractions of the parasites while the ~32-kDa PfA-M1 [1-30]-GFP fusion protein is strictly insoluble (lane 5). Note that free soluble GFP is produced from PfA-M1-GFP chimera (lane 2), but not from PfA-M1 [1-30]-GFP chimera (lane 3). Mw markers in kDa are in lane 1. **B**. Live imaging of FcB1 parasites transfected with a construct allowing expression of PfA-M1 [1-30]-GFP chimera. Top row: ring stages; bottom row: young schizont stages. Nuclei were stained with Hoechst 33342, 4 μg.ml^-1^. cc stands for "colour combine". Scale bar, 5 μm. **C**. Immuno-fluorescence analysis of FcB1 parasites transfected with a construct allowing expression of PfA-M1 [1-30]-GFP chimera, fixed by using 3.7% formaldehyde and analysed as previously described [4,6] by using rabbit anti-PfBip [21](red) and mouse anti-GFP (green) antibodies. Top row: trophozoite stages; bottom row: schizont stages. Nuclei were stained with Hoechst 33342, 4 μg.ml^-1^. cc stands for "colour combine". Scale bar, 5 μm.

In order to test whether the N-terminal segment of PfA-M1 behaves as a signal peptide and has the capacity to target proteins to the ER, this region was fused to a reporter GFP protein using the Multisite Gateway™ technology system developed by Van Dooren *et al *[27] for *Plasmodium*. Transfectant parasites express the expected ~32-kDa fusion protein that is entirely associated with the parasite membrane fraction (Figure 4A). Interestingly, no soluble GFP was cleaved off this construct (Figure 4A). Live imaging on parasites transfected with this PfA-M1 [1-30]-GFP construct revealed a pattern characteristic of ER labelling [27,33] (Figure 4B) that was confirmed by immuno-fluorescence (Figure 4C). These results strongly suggest that the N-terminal segment of PfA-M1 has the capacity to target the protein to the ER of the parasite. But in the absence of the downstream PfA-M1 sequence, this protein remains blocked in the ER and attached to the membrane.

### Determination of p96 localization depends on the concentration of saponin

**Figure 5:**
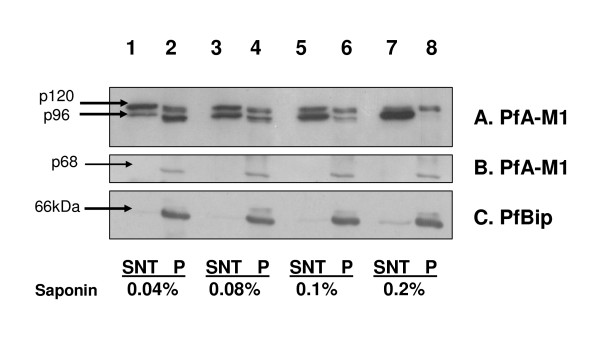
**Increasing the concentration of saponin frees p96 but not p68 from pellet to supernatant**. Infected erythrocytes at the trophozoite stage were treated with increasing concentrations of saponin from 0.04% (lanes 1 and 2), 0.08% (lanes 3 and 4), 0.1% (lanes 5 and 6) to 0.2% (lanes 7 and 8), separated into supernatant (SNT, lanes 1, 3, 5, 7) and pellet (P, lanes 2, 4, 6, 8) fractions and immunodetected with anti-MAP1 antibodies [6](A, B). Antibodies against PfBip [21] were used as controls (C). The amount of p68 (B) and PfBip (C) in the pellet fraction (parasite) remains constant with increasing concentrations of saponin while the amount of p96 released into the supernatant fraction increases with the saponin concentration (A).

To further investigate possible behavioural differences between the three forms of PfA-M1, two series of experiments were performed using synchronized trophozoite stage parasites. In a first experiment, identical numbers of iRBC were incubated with increasing concentrations of saponin (using 0.04%, 0.08%, 0.1% and to a maximum of 0.2%) and analysed to detect the three forms of PfA-M1 (Figure 5). The amount of p120 form that was released into the saponin supernatant - presumably from the PV - remained constant while that of released p96 progressively increased (see Figures 5A, B). At the maximum concentration of 0.2% saponin, p96 was exclusively fractionated to the supernatant fraction. Therefore, at low saponin concentration p96 remains associated with the parasite fraction, but as the concentration of saponin is increased, p96 shifts to the supernatant fraction corresponding to the PV. Upon similar treatment, only a minor proportion of Bip, the ER marker [21], was released to the supernatant (Figure 5C).

### Relative amounts of internal and secreted p120 and p68

**Figure 6:**
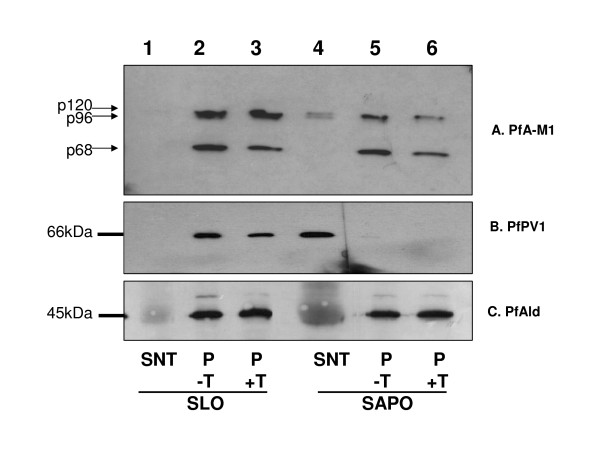
**Effect of trypsin on p120 and p68**. Infected erythrocytes at the trophozoite stage were treated with either SLO (lanes 1, 2, 3) or 0.1% saponin (SAPO) (lanes 4, 5, 6) and separated into supernatants (SNT, lanes 1 and 4) and pellets (P). Pellets were treated (P+T, lanes 3 and 6) or not (P-T, lanes 2 and 5) with 500 μg.ml^-1 ^trypsin for 30 min at room temperature and the reaction was stopped by using trypsin inhibitor (1mg.ml^-1^) for 5 min on ice before separation of samples by SDS-PAGE. Anti-MAP1 antibodies [6]were used to detect PfA-M1 forms, indicated on the left (A) or PfPV1 (B) and PfAldolase (C) as controls. The amount of p120 relative to p68 was strikingly reduced in SAPO pellets as compared to SLO pellets, after trypsin treatment.

In a second experiment, trypsin was added to iRBC after fractionation with either SLO or saponin prior to separation by SDS-PAGE and immunoblotting using anti-MAP1 PfA-M1 antibodies (Figure 6). The trypsin treatment of saponin-lysed iRBC significantly decreased the amount of p120 compared to the reduction observed for the p68 form. In addition, the trypsin treatment after SLO permeabilization (that leaves the PVM intact) does not affect the amounts of the p120 protein (nor that of p68) in the pellet fraction. Densitometric scanning indicated that ~57% of p120 is in the PV and ~43% is internal as compared to the control PfAldolase (82% internal, 18% in the PV, Figure 6C).

### Is PfA-M1trafficked back in the digestive vacuole?

**Figure 7:**
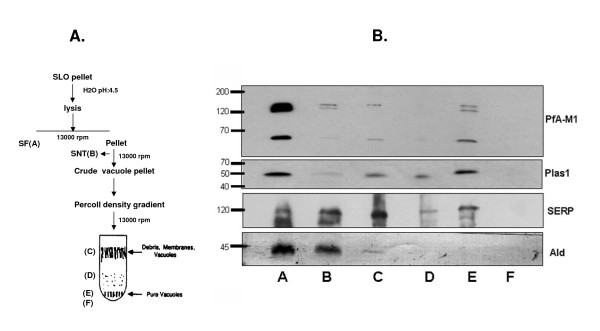
**PfA-M1 is minimally associated with FV fractions**. **A**. *P. falciparum *food vacuole fractions were isolated as shown in the schematic diagram and in the Methods section. **B**. Fractions A to F were analysed for the presence of marker proteins. Equivalent amounts of proteins were loaded for each fraction (corresponding to 4 × 10^7 ^iRBC per lane), and immunoblotted with anti-MAP1 antibodies [6] to reveal PfA-M1, or antibodies against Plasmepsin 1 [23](Plas1), PfSERP[18] (SERP) and PfAldolase [15] (Ald) as controls. The numbers on the left indicate Mw in kDa. The densitometric analysis of these immunoblots is presented as Table 1.

PfA-M1 has been recently reported to be delivered to the food vacuole (FV) [26]. This result was obtained by fusing PfA-M1 to the Yellow Fluorescent Protein (YFP) in stably transfected parasites. Some yellow fluorescence was indeed somewhat associated with the FV in these transfected parasites and some fluorescence was also associated with nuclei [26]. Experiments were designed to test whether endogenous PfA-M1 was, indeed trafficked to the FV, and specifically in which form. Parasites were fractionated as previously described [3,20] (Figure 7A) and fractions were immuno-detected with antibodies specific for PfA-M1 and three control marker proteins (Figure 7B). In these analyses, the majority of PfPlasmepsin I, a specific marker of the FV [23] was indeed associated to the pure FV fraction (fraction E, Figure 7B) as well as some PfA-M1. Densitometric measurements indicated that only 16% of the p68 form of PfA-M1 was however associated with this FV fraction, together with traces of p120 and p96 forms, while 40% of Plasmepsin I was associated with this fraction (Table 1). In this assay, the majority of PfA-M1 was associated with the soluble fractions, mainly fraction A. PfAldolase, a cytoplasmic parasite marker [15], was also mainly associated with this soluble fractions, while PfSERP, a protease associated to the parasitophorous membrane [18], was as expected mainly associated with fraction C corresponding to membranes, vacuole, and debris. From this data, it was deduced that only a minority of PfA-M1, in the p68 form, was indeed targeted to the FV, with the majority of PfA-M1 being targeted to other locations inside the parasite. Interestingly these values are in close agreement with the data by Dalal and Klemba who reported a 20% enrichment of the enzymatic activity associated with PfA-M1 in enriched FV fractions [26].

**Table 1:** Densitometric quantification of PfA-M1 forms and control proteins

fraction	p120	p96	p68	PlasI	SERP	Aldolase
**A**	56%	58%	49%	23%	15%	53%

**B**	10%	9%	8%	7%	25%	25%

**C**	9%	7%	10%	14%	29%	7%

**D**	7%	8%	9%	10%	9%	5%

**E**	10%	10%	16%	40%	17%	5%

**F**	8%	8%	8%	6%	5%	6%

The p120, p96 and p68 forms of PfA-M1 as compared to Plasmepsin I (PlasI), SERP and Aldolase were quantified in the six fractions of the FV isolation procedure. Fractions are as described on Figure 7A, i.e. fractions A and B corresponding to soluble proteins, fraction C to membranes, vacuoles, debris and fraction E to pure FV. Bands intensities were obtained by scanning each column of the Western Blot on Figure 7B. Values are expressed as a % of the sum of intensities in the six fractions for a given protein.

**Figure 8:**
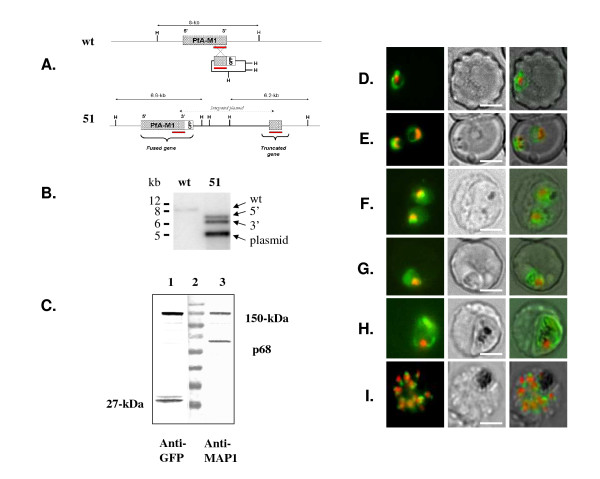
**Live imaging of FcB1 parasites transfected with a construct allowing expression of PfA-M1-GFP chimera confirms its marginal association with the FV**. **A**. Schematic representation of the integration of the PfA-M1-GFP plasmid at the PfA-M1 locus in the *P. falciparum *genome. The genomic environment of the PfA-M1 gene (MAL13P1.56) in wild type parasites (wt) is draw to scale with indication of the two flanking *Hind*III sites (H). Integration of the PfA-M1-GFP plasmid creates a fused gene and a truncated gene in transfected parasites (51). The red bar corresponds to the 1024-bp fragment encoding the C-terminal part of PfA-M1 that was cloned in the plasmid and subsequently used as probe on Southern blots. **B**. Nuclear DNA from wild type (wt) or transfected parasites (51) was digested by using *Hind*III, separated on 0.7% agarose gels in 1×TBE, transferred to nylon and hybridized with the [^32^P]-labelled 1024-bp fragment as previously described [6]. The observed pattern indicates, as expected, a wild-type *Hind*III fragment (Wt, ~8-kb) that is replaced, in transfected parasites (51), by a 5'-fused gene (~6.8-kb), a 3'-truncated gene (~6.2-kb) and a plasmid (~5-kb) *Hind*III bands. Note that this pattern is similar to that obtained by Dalal and Klemba [26]. **C**. Soluble proteins from transfected parasites (51) were separated by SDS-PAGE, transferred to nitrocellulose and immunoblotted with anti-GFP (lane 1) or anti-MAP1 [6](lane 3) antibodies as described in the Methods procedures section, revealing the recombinant PfA-M1-GFP protein, at ~150-kDa and some free GFP. Mw markers in kDa are in lane 2. Mw markers are as described on Figure 4. **D **to **I**: live imaging of transfected parasites showing: D, E. ring stages. F, G, trophozoite stages; H. young schizont. I. segmenting schizont. First column: GFP (green) and nuclei (Hoechst 33342 staining, 4 μg.ml^-1^); second column: phase contrast; third column: overlay. Scale bar, 5 μm.

This marginal delivery of PfA-M1 to the FV was further investigated by live imaging of erythrocytic stages of *P. falciparum *stably expressing a PfA-M1 protein fused to GFP (Figure 8, A to 8C) by following the procedure described by Klemba *et al *for Plasmepsin II [24]. Live parasite imaging confirmed the expression of PfA-M1 GPF chimera in rings, trophozoites and schizonts (Figure 8, D to 8I). In ring stages, the GFP fluorescence was observed as a circle surrounding the nucleus (Figure 8D, E); in trophozoites only, some GFP fluorescence was associated with FV (8G) or more frequently the nucleus (8F) as described by Dalal and Klemba [26]. In young schizonts, the GFP fluorescence associated with the FV compartment was very faint (Figure 8H). Interestingly, in segmenting schizonts, strong spots were clearly associated with and between nuclei. GFP fluorescence was absent from the FV (Figure 8I). These images are strikingly different from the images obtained in parasites expressing plasmepsin II fused to GFP in which the GFP fluorescence was strongly associated with the FV compartment [24]. It appears, therefore, that a proportion of the processed PfA-M1 is delivered to the FV only at trophozoite stage.

## Discussion

The current work is aimed at elucidating behavioural and localization differences between the different forms of PfA-M1 as a means to better understand the role(s) of this enzyme in the *P. falciparum *biology, which may shed new light on PfA-M1 biosynthesis and maturation. The three p120, p96, and p68 soluble forms of the enzyme were confirmed by using an antibody directed against the enzyme active site. Using the same antibody, a similar pattern was observed in *Plasmodium berghei *and *Plasmodium yoelii *soluble protein extracts strongly suggesting conserved maturation processes for this enzyme in the *Plasmodium *genus (I. Florent, personal communication).

Among these three forms, p120 is the only one that is found, in part, associated with membrane fractions. These experimental results fit with the predictions from the PfA-M1 gene structure: i) both Signal-P [8,10,11] and PSORT [9,37] predict a single transmembrane region located at the N-terminus of the mature peptide: ii) previous studies indicating that p96 and p68 are indeed devoid of this N-terminal region [4,6]. While Signal-P predicts that this region does not correspond to a typical eukaryotic *sensu stricto *signal peptide - it is apparently devoid of cleavage site - PSORT clearly indicates a classical signal peptide that would encompass the first 30 amino acids (Additional file 3). The N-terminal region has the capacity to drive a downstream protein to the ER where it remains attached, presumably to the ER membrane (see the results studying PfA [1-30]-GFP chimera, Figure 4A, lanes 3 and 5), while the fusion of the complete PfA-M1 to GFP does not prevent the cleavage of this N-terminal region. Therefore, some soluble PfA-M1-GFP chimera is being produced in transfected parasites (see Figure 4A lane 2).

### The p120 form of PfA-M1 targets the enzyme to the PV via ER and Golgi

Brefeldin A [34,35] which blocks protein trafficking in *P. falciparum *for both the classical secretory pathway [33] and the alternate pathway for proteins destined to the host cell [31,32] was able to block PfA-M1 under the p120 form in a parasite compartment close to the nucleus that could correspond to the ER. Because this p120 form is mainly soluble and no peptides corresponding to the N-terminal hydrophobic region could be found by the mass spectrometry analysis of PfA-M1 in the PV, it appears likely that p120 would be cleaved off this peptide during its transport through ER/Golgi towards the PV. The PV is believed to be the default pathway of plasmodial proteins having an N-terminal signal peptide [33]. The results obtained with the PfA-M1 [1-30]-GFP chimera indicate that the cleavage of this PfA-M1 signal peptide would occur at or downstream of position 30. The molecular structure and biological function of the N-terminal domain of PfA-M1 is currently being further analysed by using GFP-fusions strategies.

### The p96 form of PfA-M1 is an endogenous form of the enzyme that resides mainly outside of the parasite

The p96 form, thought until now to be an "*in vitro *degradation product" of the p120 form [4], was discovered in this current work to be a endogenous form of the enzyme that resides mainly outside the parasite in the PV and also in vesicles lysed by high concentrations of saponin. These properties of p96 are in total agreement with previously published data in which PfA-M1 biochemical studies were focused on parasites isolated by using 0.2% saponin and extensively washed in presence of protease inhibitors [4]. Indeed, in these studies, both the PV compartment and the vesicles sensitive to saponin were discarded explaining why p96 was not detected. The discovery that p96 is indeed an endogenous form of PfA-M1 provides the "missing link" between p120 and p68 forms of PfA-M1 that was extensively studied but never identified [4], and also provides a better understanding of how PfA-M1 is expressed, trafficked, and matured in parasites. Importantly, it must be emphasized that the p96 form of PfA-M1 is truly a labile form of the enzyme that is difficult to characterize biochemically. In these studies, the amount of p96 relative to p120 and p68 varied from one assay to the next, depending on the concentration of saponin used to isolate the parasite and also the age and stage distribution of these parasites.

### The p96 form of PfA-M1 is found within vesicles

**Figure 9:**
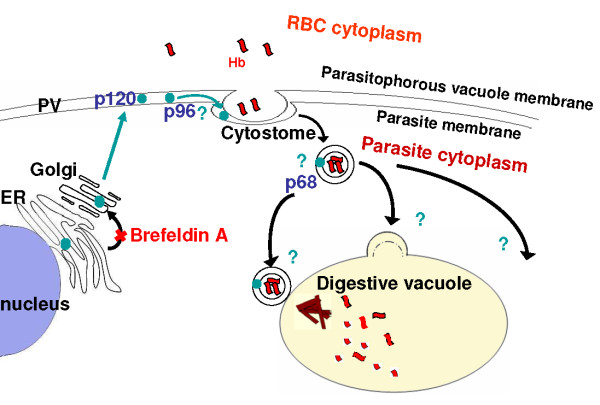
**Proposed model for the trafficking and maturation of PfA-M1 in infected erythrocytes**. PfA-M1 is targeted to the secretory pathway and the trafficking to the Golgi is sensitive to brefeldin A. The p120 is secreted into the vacuolar space where it undergoes proteolytic processing to yield the p96 form which is endocytosed within the double membrane of the cytostome and taken back into the parasite. The p96 is further processed to the p68 form which is targeted towards the digestive food vacuole, where it is marginally delivered.

In contrast to p120, p96 is within a compartment that is disrupted by higher concentrations of saponin at the periphery of the parasite and the PV. Based on published electron microscopic and ultrastructure data on *P. falciparum*, p96 could be present either in vesicles being formed at the level of the cytostome or in double membrane structures that have been previously described [38]. A logical hypothesis is that, after its delivery to the PV as the soluble p120 form, PfA-M1 is processed into the soluble p96 that returns into the parasite inside vesicles where the p96 to p68 conversion would occur, since p68 is strictly found within the parasite (Figure 9). Notably, 10,000 g and 100,000 g centrifugations of parasite extracts have no impact on the distribution of the different forms of PfA-M1 between soluble and insoluble fractions (I. Florent, personal communication). As compared to p96, p68 is devoid of the C-terminal part of PfA-M1 that has been mapped from position ~803 to the end of the protein based on Mw predictions from protein sequences (see Additional file 1). Very interestingly, a peptide of about 30 kDa corresponding closely to this C-terminal part (77% coverage from position 807 to 1085 of PfA-M1) was identified in a proteomic study recently performed to identify *Plasmodium *proteins specifically oxidized after chloroquine treatments of parasites [39]. This experimental result indicates that the C-terminal end of PfA-M1, presumably removed during the p96 to p68 conversion of PfA-M1, is indeed produced in parasites at least as an oxidized form in chloroquine-treated schizonts. The parasite compartment in which this oxidation occurs is not yet identified and will be the focus of future studies. However, this compartment appears distantly related to the FV since none of the known FV haemoglobinases were identified as being oxidized in this proteomic analysis [39]. Another outstanding question is whether the C-terminal part of PfA-M1 that is cleaved from p96 to yield p68 contains signals necessary for the return of p96 into the parasite, before its conversion under the p68 form. C-terminal signals have recently been identified in *P. falciparum *proteins, such FCP protein [40], and it has been previously noticed that the C-terminal end of PfA-M1 displays the signature of "microbodies targeting signals" [4]. This hypothesis is currently under study in the laboratory using GFP chimeras.

### What is then the final destination of PfA-M1 and what is its role in the parasite biology?

While p120 and p96 therefore appear as obligatory intermediate forms allowing proper targeting of p68 in the parasite, the enigma remains as to where p68, generated in vesicles from the p96 form, is targeted in the parasite and which function it performs. As previously mentioned, the p68 form corresponds, in terms of size and domain, to the smallest catalytic domain of M1 family metallopeptidases [4,41] and was experimentally shown to be enzymatically active [4]. The hypothesis that PfA-M1, encoded by a gene conserved through evolution from bacteria to humans [6] has been retained by the parasite exclusively to perform the function of a haemoglobinase is currently challenged by series of biological data, even if this may be one of its roles [4,6,26,42]. Beside the fact that native PfA-M1 has never been observed in FV by using immuno-fluorescence microscopy [4,6], in this current study, it does not segregate to any significant extent with pure FV fractions that do contain Plasmepsin I, a typical endoprotease of this compartment [23]. In addition, the pH at which PfA-M1 is active, which was measured experimentally, is definitively not acidic [4] (Additional file 4). An hypothesis that remains compatible with the observations by Dalal and Klemba [26] is that PfA-M1 could be directed toward the FV, but would stop at its border [4], maybe in the cytostomal vesicles that have recently been shown to have a neutral pH [43]. These results have important implications regarding the role that PfA-M1 could perform in the final steps of haemoglobin digestion recently documented by using inhibitors [42]. Indeed, rather than being present on the cytoplasmic face of the FV, as recently proposed [42,44] and discussed [45], the current study suggests a presence of PfA-M1 within vesicles. Another possibility is that PfA-M1 would be directly involved in vesicular trafficking from the PV towards the FV. In fact, in mammals and plants some M1 family members have been shown to be involved in vesicular trafficking. For example, IRAP (Insulin-regulated glucose transporter) mediates the trafficking of vesicles laden with glucose to the plasma membrane [46]. Alternatively, PfA-M1 could be targeted to many other destinations, such as the nucleus, as recently proposed by Dalal and Klemba [26] or even have pleiotropic roles as found for example for falcilysin [47]. Importantly, it must be noted that the series of transcriptomic and proteomic studies performed on *P. falciparum*, rodent malaria, and bloodstream and insect stages all converge towards the fact that PfA-M1 expression is not at all restricted to bloodstream stages [48-52]. In particular, PfA-M1 was detected in *P. falciparum *gametocytes and gametes [51], in *P. falciparum *sporozoites [49,52], and PbA-M1, the orthologue PfA-M1 in *P. berghei *was also detected in *P. berghei *gametocytes and ookinetes [50,53]. Very recently, PfA-M1 was identified by mass-spectrometry in sporozoites derived from oocysts and in sporozoites isolated form salivary glands [52]. Taken together, these results strongly suggest the role played by PfA-M1 in the parasite biology is probably not restricted to haemoglobin digestion in or outside the FV. Deciphering the complexity of its trafficking and maturation in infected red blood cells or other parasite stages is, therefore, of key importance for the further development of efficient inhibitors against this enzyme [42,54,55].

## Conclusion

In the current study, new biochemical data allowed to biologically differentiate between the three forms of PfA-M1, p120 being a precursor form of PfA-M1 likely found in ER, Golgi and PV, p96 being a transient form located in PV and vesicles, and p68, the final processed form being yielded in vesicles likely trafficking back from the PV to the parasite since p68 is exclusively localized within the parasite. These results bring new insights regarding PfA-M1 topology and relation to key compartments of the infected red blood cell in particular relative to the food vacuole. Indeed, PfA-M1 would reside in the ER, Golgi and PV and also outside the food vacuole but inside vesicles, or be marginally delivered into the food vacuole at trophozoite stages, but would not be in direct contact with the parasite cytoplasm, contrary to the recent proposal that the membrane form of PfA-M1 would be attached on the cytoplasmic side of the food vacuole [44]. Such a localization of PfA-M1 has, therefore, important implications both for its biological function and for further improvement of established inhibitors against this putative therapeutic target [42,54,55].

## Abbreviations

AMC: 7-amido-4-methylcoumarin; BFA: brefeldin A; DIGE: Difference In Gel Electrophoresis; ER: endoplasmic reticulum; FV: food vacuole; GFP: green fluorescent protein; iRBC: infected red blood cells; MAP: multiple antigenic peptide; PfA-M1: *Plasmodium falciparum *aminopeptidase M1; PSORT: prediction of protein sorting signals; PV: parasitophorous vacuole; PVM: parasitophorous vacuole membrane; SAPO: saponin; SLO: streptolysin O; YFP: yellow fluorescent protein.

## Competing interests

The authors declare that they have no competing interests.

## Authors' contributions

OA and JN performed experimental biochemical work including saponin and streptolysin fractionations, food vacuole fractionations, and mass spectrometry. CS and IF performed 2D-gel analyses, BFA studies, and constructed transfectants expressing PfA-M1-GFP chimera. CS, IF and MG performed the imaging. JN and IF wrote the paper. All authors have read and approved the final manuscript.

## Supplementary Material

Additional file 1 **Structure of the full length PfA-M1 and of the three p120, p96 and p68 forms**. The full length PfA-M1 (top line) is as predicted from the gene structure [6] and EMBL Y09081.2. It starts with a 24 amino-acids N-terminal hydrophobic domain which is a putative signal sequence (SS) and terminates with a putative microbodies targeting signal (NKL) [4,6]. The N and C-termini of p96 and p68 forms are not clearly defined but their N-termini have been experimentally shown to be located between the MAP2 (^141^SDKMKPYEEGHG^153^) and MAP1 (^192^KNEPKIHYRKDYK^205^) epitopes [4]. The three p120, p96 and p68 forms are soluble and all contain a full active site (canonical sequence [GAMEN]-[HExxHx18E]-[Y], [4,41]). The arrow below p68 corresponds to the PfA-M1 domain expressed as recombinant protein (amino acids 191 to 802). Click here for file

Additional file 2 **anti-p68 and anti-MAP1 antibodies label the same proteins in parasite extracts**. Soluble parasite extracts from asynchronous cultures were separated by 2D-gel electrophoresis before western transfer and immunodetection using anti-p68 antibodies (A) and anti-MAP1 antibodies (B), revealing identical patterns. The ladder above the gels represents the pH3-pH10 strips used for isoelectricfocussing, with the various isoelectric points. Molecular weight markers, on the left of each gel are in kDa. Arrows point to the p120, p96 and p68 forms of PfA-M1, respectively. Click here for file

Additional file 3 **Conflicting results are yielded by Signal-P and PSORT analyses. A**. PfA-M1 sequence was analyzed by Signal-P by using "eukaryotic sequence" setting; the output clearly indicates the presence of a 24 amino acids hydrophobic domain (S-score) but the absence of a typical cleavage site (C and Y scores). **B**. PfA-M1 was analysed by PSORT http://www.psort.org/ and a classical signal peptide encompassing the 30 first amino acids was predicted. Click here for file

Additional file 4 **pH dependency of PfA-M1 enzymatic activity**. PfA-M1 activity (pure native enzyme and L-Leu-AMC substrate) was measured between pH 4.0 and pH 10.0 and the results are plotted as % of maximal activity (pH 7.5). This diagram corresponds to a graphical representation of the experimental data by [4]. Click here for file
