# Supplementary figures and images for: Plasmodium falciparum PfA-M1 aminopeptidase is trafficked via the parasitophorous vacuole and marginally delivered to the food vacuole
Source: Malar J. 2010 Jun 30;9:189. doi: 10.1186/1475-2875-9-189 (PMC2914058; doi:10.1186/1475-2875-9-189)

## Additional File 1

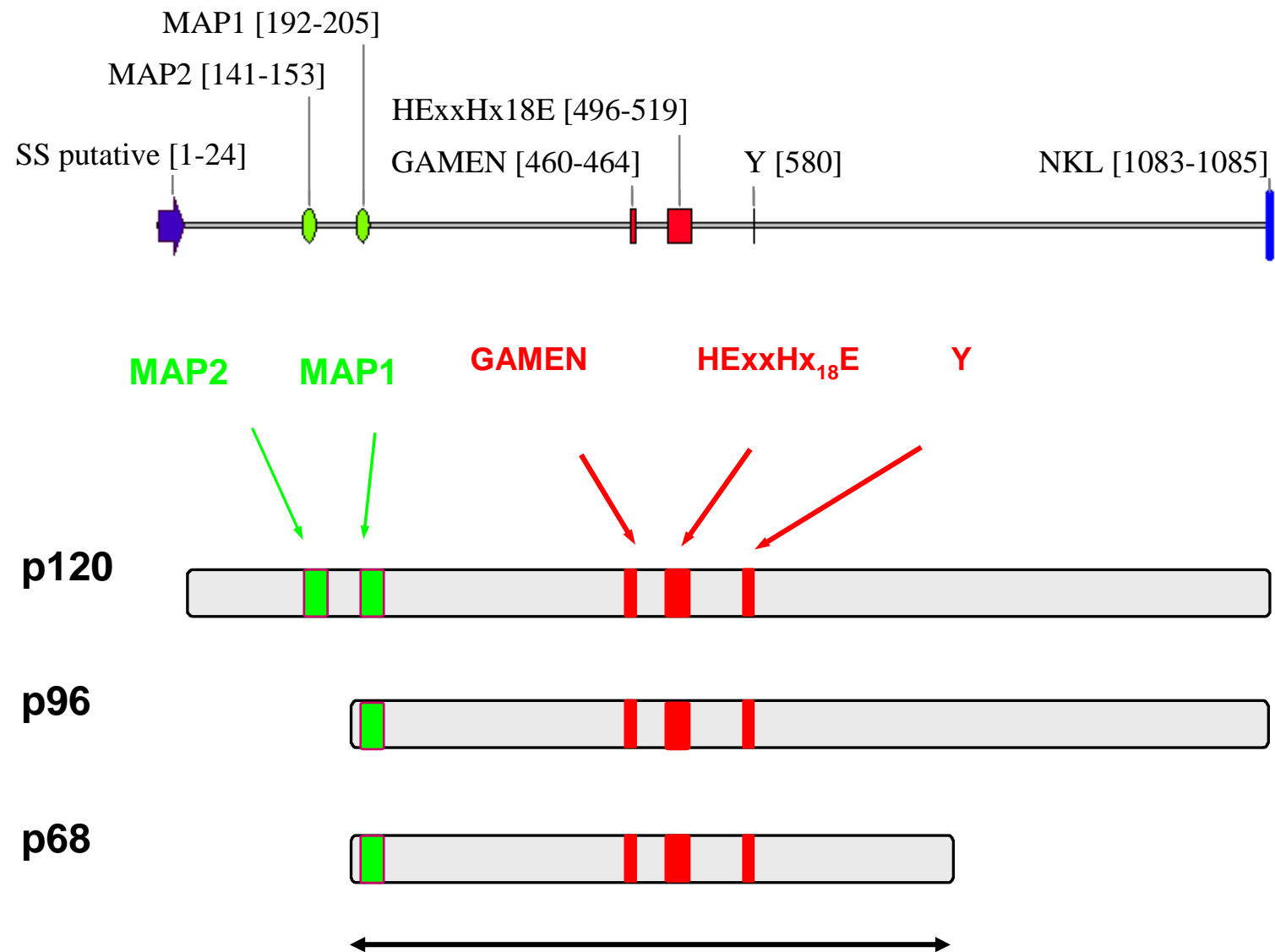

Supplement: Additional file 1 — Structure of the full length PfA-M1 and of the three p120, p96 and p68 forms. The full length PfA-M1 (top line) is as predicted from the gene structure [6] and EMBL Y09081.2. It starts with a 24 amino-acids N-terminal hydrophobic domain which is a putative signal sequence (SS) and terminates with a putative microbodies targeting signal (NKL) [4,6]. The N and C-termini of p96 and p68 forms are not clearly defined but their N-termini have been experimentally shown to be located between the MAP2 (141SDKMKPYEEGHG153) and MAP1 (192KNEPKIHYRKDYK205) epitopes [4]. The three p120, p96 and p68 forms are soluble and all contain a full active site (canonical sequence [GAMEN]-[HExxHx18E]-[Y], [4,41]). The arrow below p68 corresponds to the PfA-M1 domain expressed as recombinant protein (amino acids 191 to 802). [file 1475-2875-9-189-S1.PDF]

## Additional File 2

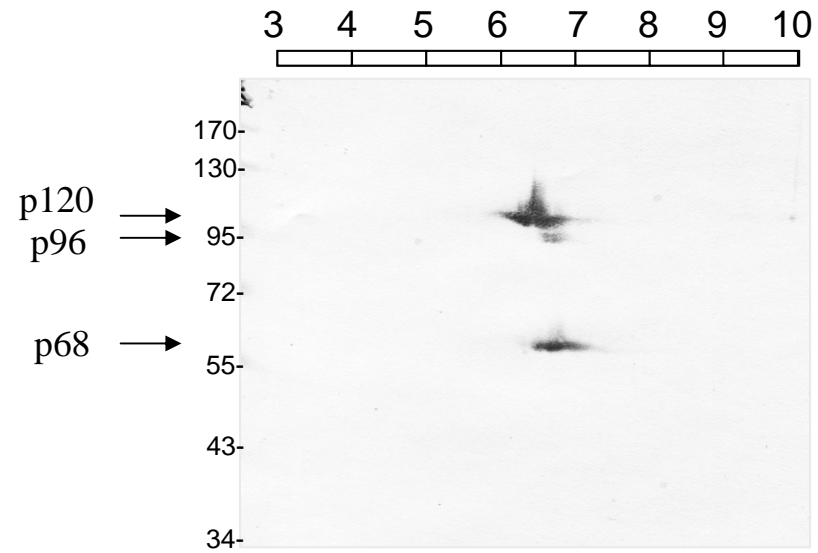

A. Anti-p68

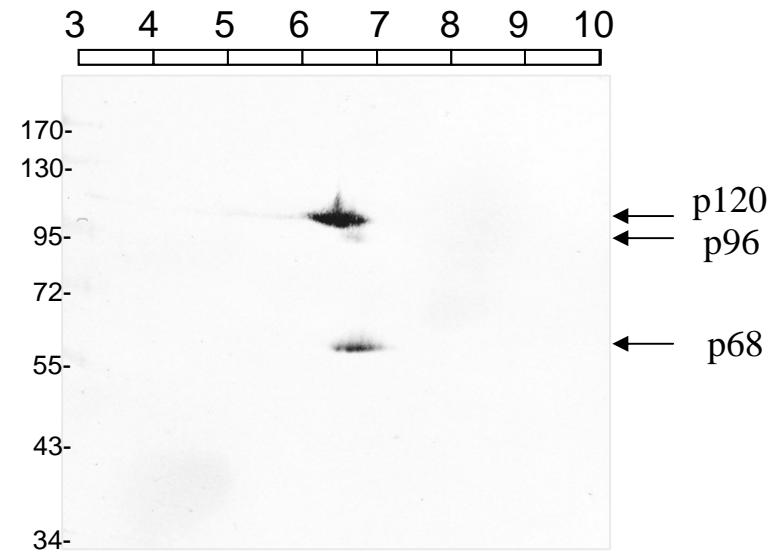

B. Anti-MAP1

Supplement: Additional file 2 — anti-p68 and anti-MAP1 antibodies label the same proteins in parasite extracts. Soluble parasite extracts from asynchronous cultures were separated by 2D-gel electrophoresis before western transfer and immunodetection using anti-p68 antibodies (A) and anti-MAP1 antibodies (B), revealing identical patterns. The ladder above the gels represents the pH3-pH10 strips used for isoelectricfocussing, with the various isoelectric points. Molecular weight markers, on the left of each gel are in kDa. Arrows point to the p120, p96 and p68 forms of PfA-M1, respectively. [file 1475-2875-9-189-S2.PDF]

## Additional File 4

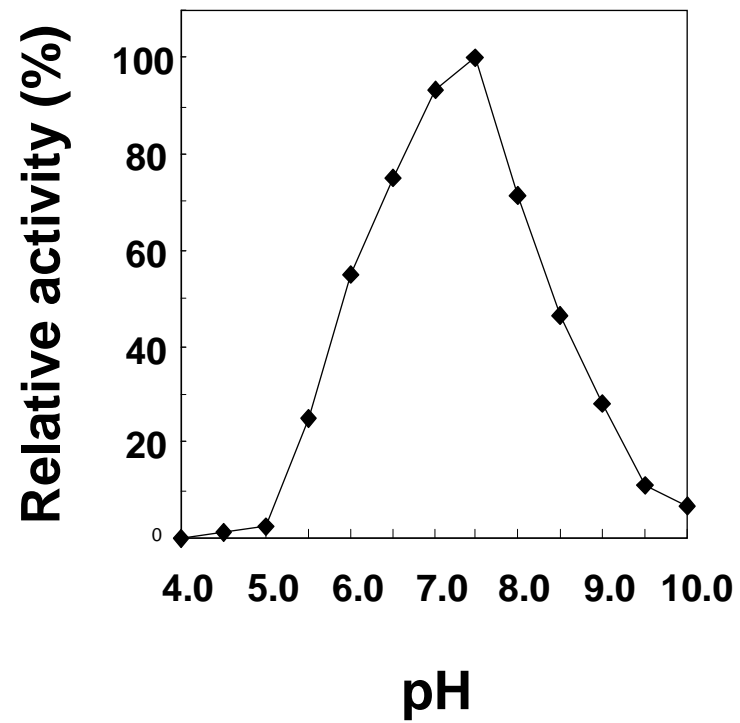

Supplement: Additional file 4 — pH dependency of PfA-M1 enzymatic activity. PfA-M1 activity (pure native enzyme and L-Leu-AMC substrate) was measured between pH 4.0 and pH 10.0 and the results are plotted as % of maximal activity (pH 7.5). This diagram corresponds to a graphical representation of the experimental data by [4]. [file 1475-2875-9-189-S4.PDF]
