# Supplementary material for: Plasmodium falciparum PfA-M1 aminopeptidase is trafficked via the parasitophorous vacuole and marginally delivered to the food vacuole
Source: Malar J. 2010 Jun 30;9:189. doi: 10.1186/1475-2875-9-189 (PMC2914058; doi:10.1186/1475-2875-9-189)
Supplement: Additional file 3 — Conflicting results are yielded by Signal-P and PSORT analyses. A. PfA-M1 sequence was analyzed by Signal-P by using "eukaryotic sequence" setting; the output clearly indicates the presence of a 24 amino acids hydrophobic domain (S-score) but the absence of a typical cleavage site (C and Y scores). B. PfA-M1 was analysed by PSORT http://www.psort.org/ and a classical signal peptide encompassing the 30 first amino acids was predicted. [file 1475-2875-9-189-S3.PDF]

Additional File 3

A.

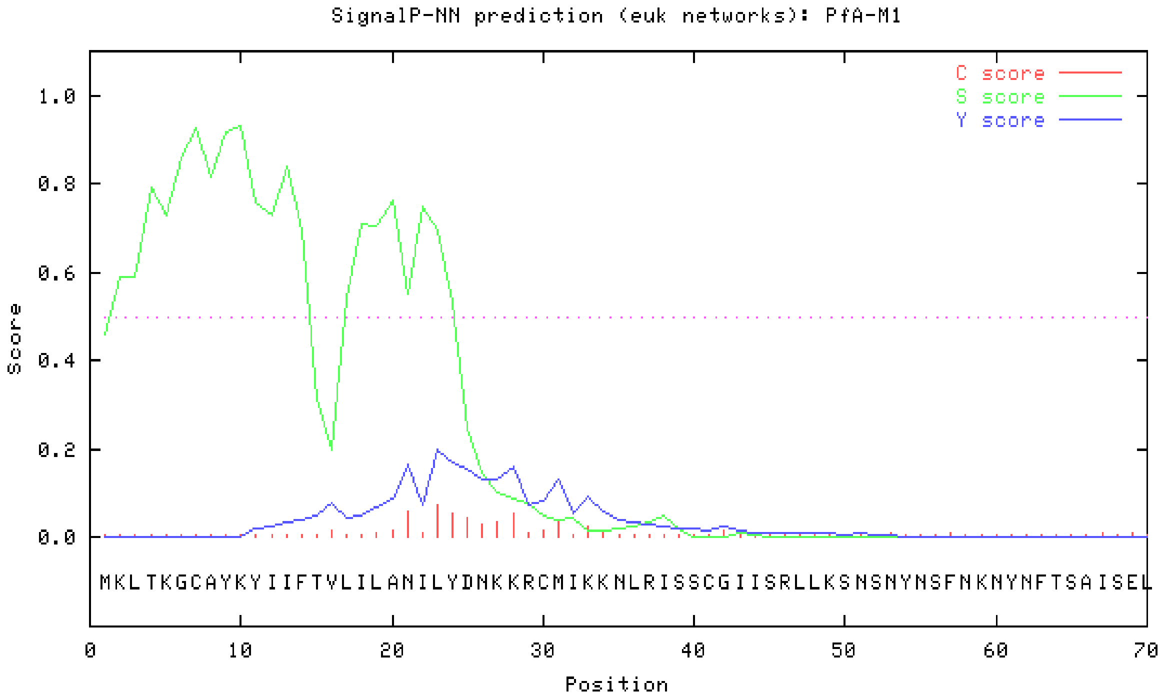

B.

iPSORT Prediction

Predicted as: *having a signal peptide*

Sequence (Type: nonplant)

1 MKLTK GCAYK YIIFT VLILA NILYD NKKRC MIKKN LRISS CGIIS RLLKS
51 NSNYN SFNKN YNFTS AISEL QFSNF WNLDI LQKDI FSNIH NNKNK PQSYI
101 IHKRL MSEKG DNNNN NHQNN NGNDN KKRLG SVVNN EENTC SDKRM KPFEE
151 GHGIT QVDKM NNNSD HLQQN GVMNL NSNNV ENNNN NNSVV VKKNE PKIHY
201 RKDYK PSGFI INNVT LNINI HDNET IVRSV LDMDI SKHNV GEDLV FDGVG
